# Supplementary material for: HIVprotI: an integrated web based platform for prediction and design of HIV proteins inhibitors
Source: J Cheminform. 2018 Mar 9;10:12. doi: 10.1186/s13321-018-0266-y (PMC5845081; doi:10.1186/s13321-018-0266-y)
Supplement: Supplementary file 2 — Additional file 2. Source code of HIVProtI web server. [file 13321_2018_266_MOESM2_ESM.zip › HIVprotI_Source-code/hivprotI/weka-3-6-6/doc/weka/core/logging/Logger.html]

Logger


---


|  |  |  |
| --- | --- | --- |
| **Overview** \| **Package** \| **Class** \| **Tree** \| **Deprecated** \| **Index** \| **Help** \| **Weka's home** | |  |
| **PREV CLASS**   **NEXT CLASS** | **FRAMES**    **NO FRAMES**     **All Classes** |  |
| SUMMARY: NESTED \| FIELD \| CONSTR \| METHOD | DETAIL: FIELD \| CONSTR \| METHOD |  |


---


## weka.core.logging Class Logger

```
java.lang.Object
  weka.core.logging.Logger
```

**All Implemented Interfaces:**: RevisionHandler

**Direct Known Subclasses:**: ConsoleLogger

---

``` public abstract class Logger extends java.lang.Object implements RevisionHandler ```

Abstract superclass for all loggers.

**Version:**
:   $Revision: 4716 $

**Author:**
:   fracpete (fracpete at waikato dot ac dot nz)

---

| **Nested Class Summary** | |
| --- | --- |
| `static class` | `Logger.Level`             The logging level. |


| **Field Summary** | |
| --- | --- |
| `static java.lang.String` | `PROPERTIES_FILE`             the properties file. |


**Constructor Summary**

`Logger()`
  
          Initializes the logger.


| **Method Summary** | |
| --- | --- |
| `Logger.Level` | `getMinLevel()`             Returns the minimum level log messages must have in order to appear in the log. |
| `static Logger` | `getSingleton()`             Returns the singleton instance of the logger. |
| `static void` | `log(Logger.Level level, java.lang.String msg)`             Logs the given message under the given level. |
| `static void` | `log(Logger.Level level, java.lang.Throwable t)`             Logs the given message under the given level. |

**Methods inherited from class java.lang.Object**

`equals, getClass, hashCode, notify, notifyAll, toString, wait, wait, wait`

**Methods inherited from interface weka.core.RevisionHandler**

`getRevision`

**Field Detail**

### PROPERTIES\_FILE

```
public static final java.lang.String PROPERTIES_FILE
```

:   the properties file.

    **See Also:**: Constant Field Values


**Constructor Detail**

### Logger

```
public Logger()
```

:   Initializes the logger.


**Method Detail**

### getMinLevel

```
public Logger.Level getMinLevel()
```

:   Returns the minimum level log messages must have in order to appear in
    the log.

    :   **Returns:**: the level

---


### getSingleton

```
public static Logger getSingleton()
```

:   Returns the singleton instance of the logger.

    :   **Returns:**: the logger instance

---


### log

```
public static void log(Logger.Level level,
                       java.lang.String msg)
```

:   Logs the given message under the given level.

    :   **Parameters:**: `level` - the level of the message: `msg` - the message to log

---


### log

```
public static void log(Logger.Level level,
                       java.lang.Throwable t)
```

:   Logs the given message under the given level.

    :   **Parameters:**: `level` - the level of the message: `t` - the throwable to log


---


|  |  |  |
| --- | --- | --- |
| **Overview** \| **Package** \| **Class** \| **Tree** \| **Deprecated** \| **Index** \| **Help** \| **Weka's home** | |  |
| **PREV CLASS**   **NEXT CLASS** | **FRAMES**    **NO FRAMES**     **All Classes** |  |
| SUMMARY: NESTED \| FIELD \| CONSTR \| METHOD | DETAIL: FIELD \| CONSTR \| METHOD |  |


---
